# Severe oxidative stress in an acute inflammatory demyelinating model in the rhesus monkey

**DOI:** 10.1371/journal.pone.0188013

**Published:** 2017-11-14

**Authors:** Jordon Dunham, Reinofke van de Vis, Jan Bauer, Jacqueline Wubben, Nikki van Driel, Jon D. Laman, Bert A. ‘t Hart, Yolanda S. Kap

**Affiliations:** 1 Department of Immunobiology, Biomedical Primate Research Centre, Rijswijk, The Netherlands; 2 University Groningen, University Medical Center, Department of Neuroscience, Groningen, The Netherlands; 3 Department Neuroimmunology, Brain Research Institute, Medical University, Vienna, Austria; San Raffaele Scientific Institue, ITALY

## Abstract

Oxidative stress is increasingly implicated as a co-factor of tissue injury in inflammatory/demyelinating disorders of the central nervous system (CNS), such as multiple sclerosis (MS). While rodent experimental autoimmune encephalomyelitis (EAE) models diverge from human demyelinating disorders with respect to limited oxidative injury, we observed that in a non-human primate (NHP) model for MS, namely EAE in the common marmoset, key pathological features of the disease were recapitulated, including oxidative tissue injury. Here, we investigated the presence of oxidative injury in another NHP EAE model, i.e. in rhesus macaques, which yields an acute demyelinating disease, which may more closely resemble acute disseminated encephalomyelitis (ADEM) than MS. Rhesus monkey EAE diverges from marmoset EAE by abundant neutrophil recruitment into the CNS and destructive injury to white matter. This difference prompted us to investigate to which extent the oxidative pathway features elicited in MS and marmoset EAE are reflected in the acute rhesus monkey EAE model. The rhesus EAE brain was characterized by widespread demyelination and active lesions containing numerous phagocytic cells and to a lesser extent T cells. We observed induction of the oxidative stress pathway, including injury, with a predilection of p22phox expression in neutrophils and macrophages/microglia. In addition, changes in iron were observed. These results indicate that pathogenic mechanisms in the rhesus EAE model may differ from the marmoset EAE and MS brain due to the neutrophil involvement, but may in the end lead to similar induction of oxidative stress and injury.

## Introduction

Cell-mediated demyelinating diseases of the central nervous system (CNS) are idiopathic disorders that include multiple sclerosis (MS) and acute disseminated encephalomyelitis (ADEM) [1]. As these diseases have a predilection for onset and diagnosis in children (ADEM) or young adults (MS), the quality of life is adversely affected during prime activity years [2]. One inherent commonality of these diseases, as well as with other neuro-immunological disorders, such as stroke, is a dysfunction of the blood-brain barrier and leukocyte extravasation from the periphery into the CNS [1, 3]. Although both the innate and adaptive arms of the immune system play a role in disease activity, the innate immune arm plays a particularly vital role in orchestrating demyelinating pathologies [4].

Non-human primates (NHP) have emerged as valuable pre-clinical models for the translation of scientific discoveries in rodent-based models of (auto)immune inflammatory diseases to clinical application [5]. NHP, compared to mice, not only have a much closer evolutionary proximity to humans, which is reflected by genetic and immunological similarity, but they also display more similar complexity of neuro-anatomical structures [6, 7]. EAE can readily be induced in diverse NHP species. Both common marmosets (*Callithrix jacchus*) and rhesus macaques (*Macaca mulatta*) are equally susceptible to developing EAE, yet they differ in their requirement for adjuvant in EAE induction and the manner in which the ensuing disease progresses [8–11]. Despite a closer biological and phylogenetic relationship of rhesus monkeys to human than marmoset, disease development in the rhesus EAE model is much more acute and aggressive.

A central question underlying the current study is why the identical disease induction protocol (rhMOG/CFA) elicits such different types of EAE in marmosets and rhesus monkeys. A characteristic pathological difference between the EAE models is the presence of large necrotic/hemorrhagic lesions in some rhesus monkeys, which are never found in the corresponding marmoset EAE models [9]. Based on clinical and pathological presentation, EAE in rhesus monkeys more closely resembles ADEM than MS; ADEM is a juvenile form of MS that displays a rapid and aggressive clinical course including symptoms such as vomiting, vision impairment, and acute neurological deficits, such as rapid onset of hemiparesis [12, 13].

In the rhesus monkey EAE model, evidence for a pathogenic role of neutrophils was found, but their exact role in the animal model has not been analysed [14]. Neutrophilic granulocytes are abundant in peripheral blood and rapidly mobilize to sites of infection or damage where they phagocytose pathogens, release proteinases and anti-microbial peptides, and form extracellular traps [4, 15]. Similar to macrophages, neutrophils generate reactive oxygen species (ROS) by electron chaperone NADPH oxidase complexes localized in phagolysosomes and cell membranes. The activation of NADPH oxidase involves the assembly of cytosolic (p47phox and p67phpox) with membrane-bound (gp91phox, p22phox) subunits into a multimeric complex [16]. While ROS have a vital role in the intra-phagosomal degradation of pathogens, they can elicit oxidative stress and oxidative tissue injury when released outside cells [17]. The brain is naturally highly vulnerable to oxidative stress, which is due to the high polyunsaturated fatty acid content of the neuronal membranes and the high oxygen consumption relative to the rest of the body [18]. Oxidative injury and mitochondrial dysfunction are now implicated as main causative factors of axon degeneration in the MS brain (reviewed in [19, 20]).

The inflammatory active MS lesion is characterized by the expression of ROS generating NADPH oxidase in resident (microglia) and infiltrating (macrophages) phagocytic cells. The presence of markers associated with oxidative stress, such as lipid peroxidation, coincided with marked upregulation of anti-oxidant enzymes, such as superoxide dismutase (SOD) 1 and 2 [21, 22]. SOD2 was found prominently expressed in astrocytes and neurons of the MS and marmoset EAE brain [21, 23, 24]. Up-regulation of mitochondrial heat shock protein 70 (mtHSP70) is a known tissue reaction to oxidative stress to minimize protein aggregation [25]. Recently, we have shown that oxidative stress and injury are key pathological features of the marmoset EAE model [26]. A recognized limitation of rodent EAE as translationally relevant model for MS is the inadequate replication of oxidative stress and injury [27]. The presence of these features in the NHP EAE brain may in part be attributed to the similar accumulation of iron observed in both marmoset and human brain. Iron may amplify oxidative injury by catalyzing the formation of highly toxic hydroxyl radicals via the Haber-Weiss reaction [28].

The aim of the current study was to characterize the activation of the oxidative stress pathway in the acute demyelinating rhesus monkey EAE model. We show abundance of NADPH oxidase expressing neutrophils, significant increase of anti-oxidants in the lesions and some changes in iron in the rhesus monkey EAE brain. We propose that just like in mouse and marmoset EAE models, lesions are formed by a combined cellular and humoral autoimmune attack causing inflammation and demyelination. When activated neutrophils releasing high amounts of oxyradicals are present in the lesions the oxidative pressure on the tissue is amplified. In the presence of iron released from oligodendrocyte/myelin complexes highly aggressive oxyradicals are formed, which can cause the necrotic aspect of the lesions.

## Materials and methods

### Rhesus monkey tissues

Cryopreserved and formalin-fixed, paraffin-embedded tissues from previous rhesus monkey EAE experiments performed at the Biomedical Primate Research Centre (BPRC, Rijswijk, The Netherlands) were used for this study. These studies were reviewed and approved by the institutional ethics review committee. EAE induction was performed by immunization with human recombinant myelin oligodendrocyte glycoprotein (rhMOG) emulsified in CFA (rhMOG/CFA). The monkeys for this study were selected based upon development of clinically evident disease severe enough to warrant humane euthanasia. For a detailed description of disease induction and monitoring see Haanstra et al [12]. Monkeys included in this study were five to eight years old (Table 1).

**Table 1:** Animals used for analysis.

	Age (y)	EAE score at euthanasia	Lesions in brain
R06030	7.2	2.5	perivascular
R06052	7.1	5	perivascular
R06088	7.0	5	perivascular
R07035	6.1	5	perivascular
R08043	5.1	5	perivascular and hemorrhagic

### Immunohistochemistry

Immunohistochemical analysis of formalin-fixed paraffin embedded material was performed on 5 μm sections. Sections were deparaffinized using xylene (VWR, Radnor, PA), rehydrated via graded ethanol into distilled water and blocked for endogenous peroxidase activity by incubating tissue in 0.03% hydrogen peroxide (Sigma, St. Louis, MO,) in methanol for 30 min. Heat-induced antigen retrieval was performed in either EDTA (pH 8.6; Sigma) or citrate (pH 6.0; Sigma) and tissue was rinsed twice in Tris-buffered saline (TBS) following steaming. To block non-specific antibody binding, tissue sections were incubated in 10% FCS in Dako wash buffer (DAKO, Glostrup, Denmark) for 30 min. To stain cryo-preserved material, sections of 6–8 μm were mounted onto permafrost plus tissue slides and fixated with acetone (10 min). Prior to nonspecific antibody blocking tissue sections were air dried for 10 min, rinsed with PBS, and incubated in PBS with 0.03% hydrogen peroxide to block endogenous peroxidase.

Tissue sections were incubated with primary antibodies (See Table 2) overnight at 4°C. Following wash steps with TBS to remove excess antibody. Biotin-labeled secondary antibodies (Jackson ImmunoResearch Laboratories, West Grove, PA) were added to tissue sections for 1 h at room temperature. Following an additional wash step, avidin-labeled peroxidase (Sigma, 1:150) was added prior to visualization with diaminobenzidine tetrachloride (DAB, Sigma). A hemalaun counterstain was performed to visualize nuclei by incubation tissue for 2 min in a 1:10 diluted hemalaun (Merck Millipore; Billerica, MA). Finally, tissue was dehydrated with graded ethanol and xylene prior to mounting with malinol (Waldeck, Münster, Germany).

**Table 2:** Antibodies used for immunocytochemistry.

Primary antibody	Company	Catalog	Host	Target
HLA-DR	DAKO	M0775	Mouse	Antigen presentation
Iba-1	Abcam	AB15690	Mouse	Microglia
MRP14	BMA biomedicals	S100A9	Mouse	Macrophage
CD3	Agilent Technologies	A0452	Rabbit	T cell
CD66	Miltenyi Biotec	130-093-133	Mouse	Neutrophil
PLP	Serotec	MCA839G	Mouse	Myelin
GFAP	Sigma	SAB5201104	Mouse	Astrocyte
NeuN	Millipore	MAB377	Mouse	Neuron
P22phox	Santa-cruz	SC20781	Rabbit	NADPH oxidase subunit
iNOS	Chemicon	AB16311	Rabbit	Reactive nitrogen species
mtHSP70	ThermoFisher	MA3-028	Mouse	Oxidative stress (mitochondria)
SOD2	Abcam	ab13533	Rabbit	Oxidative Stress (mitochondria)
8-OHdG	Abcam	ab62623	Mouse	Oxidative Injury

To quantify expression of cellular and oxidative stress markers following a gross pathological analysis of both white matter and grey matter (cortical) (mid) brain regions, 400x images of normal appearing white matter (NAWM) (5 animals) and of active white matter lesions (5 animals) were converted to 8 bit using ImageJ and a threshold was applied (to images) to eliminate non-specific staining. Standard scale bars were used to calculate the area of the images to determine cells/area and data is presented as the number of positively staining cells /mm^2^.

### Immunofluorescence

Double or triple fluorescent labeling was employed to determine co-localization of expression of various markers associated with the oxidative damage pathway. Staining was performed similar to the immunohistochemistry protocol described above, with minor deviations. Briefly, following overnight incubation at 4°C with primary antibodies diluted in Dako ready-to-use diluent (DAKO) or TBS with 10% FCS, slides were washed in TBS and incubated for an additional 1 h. For visualization, tissue was incubated for 1 h at room temperature with directly labeled secondary antibodies against primary host species conjugated to CY2, CY3 and CY5 (Jackson ImmunoResearch Laboratories). Cell nuclei were visualized using a commercially available Vectashield DAPI antifade mounting kit (Vector laboratories, Burlingame, CA). Fluorescence images were taken with a 63x oil immersion lens on a Leica systems microscope (DMI 600B; Leica Microsystems GmbH, Wetzlar, Germany).

### Iron staining

To determine the iron content of the post-mortem primate brain, DAB-enhanced TurnBull staining of non-heme tissue iron was performed on formalin-fixed paraffin-embedded tissue sections as described [29, 30]. Briefly, paraffin-embedded tissue sections of 2–3 μm thickness were deparaffinized using xylene and then rehydrated via graded ethanol into distilled water. Tissues were treated with 10% ammonium sulfide (Merk Millipore; NY) for 1.5 h and with potassium-ferricyanide (Sigma) for 15 min. Endogenous peroxidase was blocked with 0.03% hydrogen peroxide in methanol (Sigma) prior to amplification with 0.025% 3,3'-diaminobenzidine (Sigma, St. Louis, MO). Hemalaun counterstaining was performed as described above.

### Figures and statistics

Figures were made using Adobe InDesign CC 2015 (Adobe Systems, San Jose, CA) and in some cases images were adjusted for brightness using Adobe Photoshop CC 2015 (Adobe Systems). Statistics (Mann-Whitney test) was performed using Graphpad Prism 5.0 (GraphPad Software, Inc., La Jolla, CA).

## Results

### Neutrophil-mediated widespread demyelination in rhesus monkey EAE

A generic pathological characterization was performed on brains of five rhesus monkeys immunized with rhMOG/CFA-induced EAE. Perivascular lesions could be detected in brains of all monkeys and in one monkey also hemorrhagic lesions could be observed (Table 1). Demyelination was mainly perivascular, whereas inflammation was widespread. For further quantification, three areas of NAWM and three perivascular lesions of each animal were analyzed. Hemorrhagic lesions were not included in these analyses.

**Fig 1:**
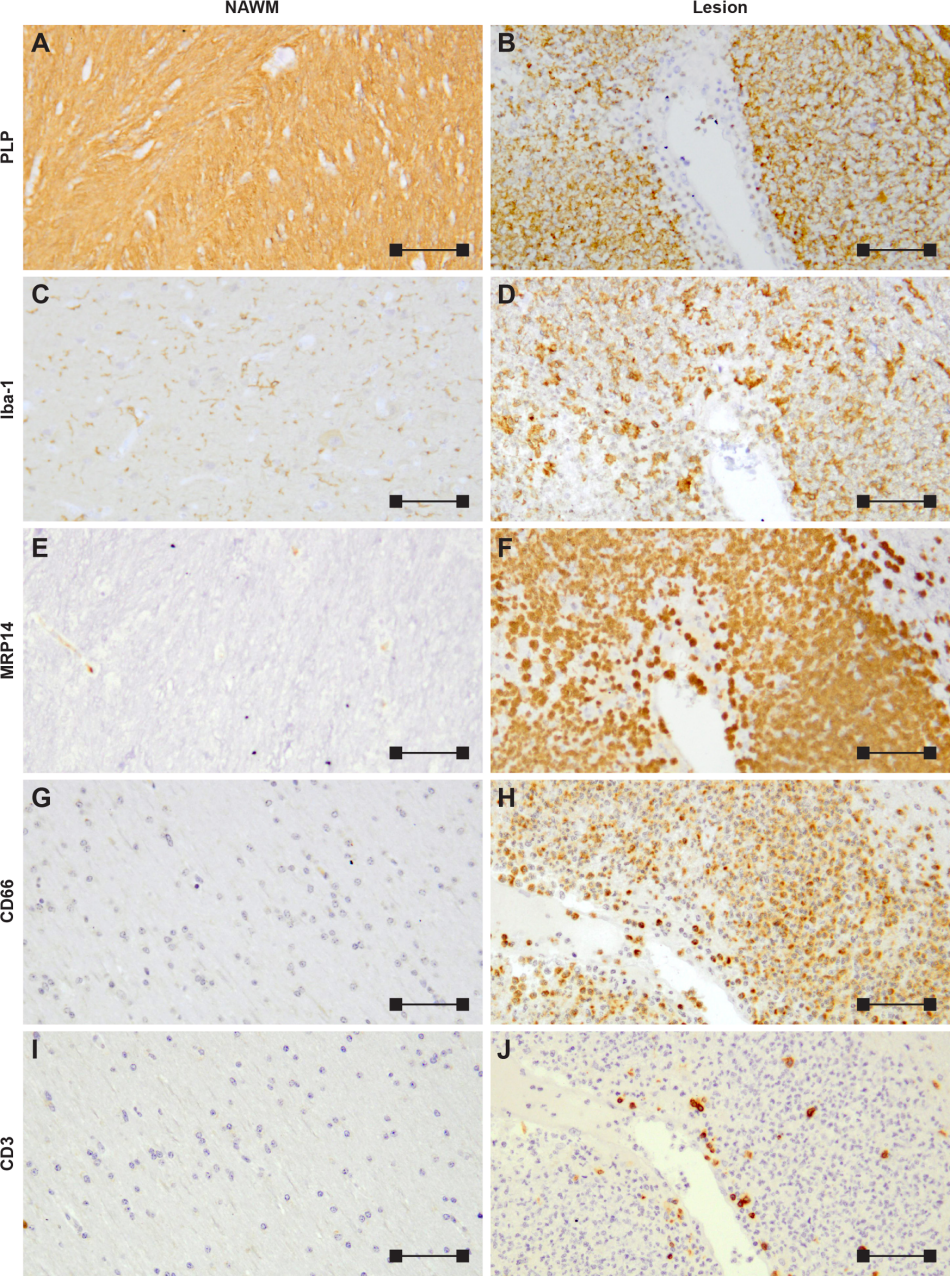
Characterization of rhesus monkey EAE brain pathology. Tissue was stained for myelin (PLP; A-B), microglia/macrophages (Iba-1; C-D), macrophages & neutrophils (MRP14; E-F), neutrophils (CD66; G-H), and T cell markers (CD3; I-J). In the NAWM (left column), infiltrating immune cells were sparse or absent, yet markedly upregulated in the active lesions (right column). The dominant immune cell types of the active lesion were macrophage/microglia (D, F) and neutrophils (H). T cells, as determined by CD3 positivity, were readily detected in large numbers, yet visually less abundant than other cell types (J). The image scale bar is 100 μm.

**Fig 2:**
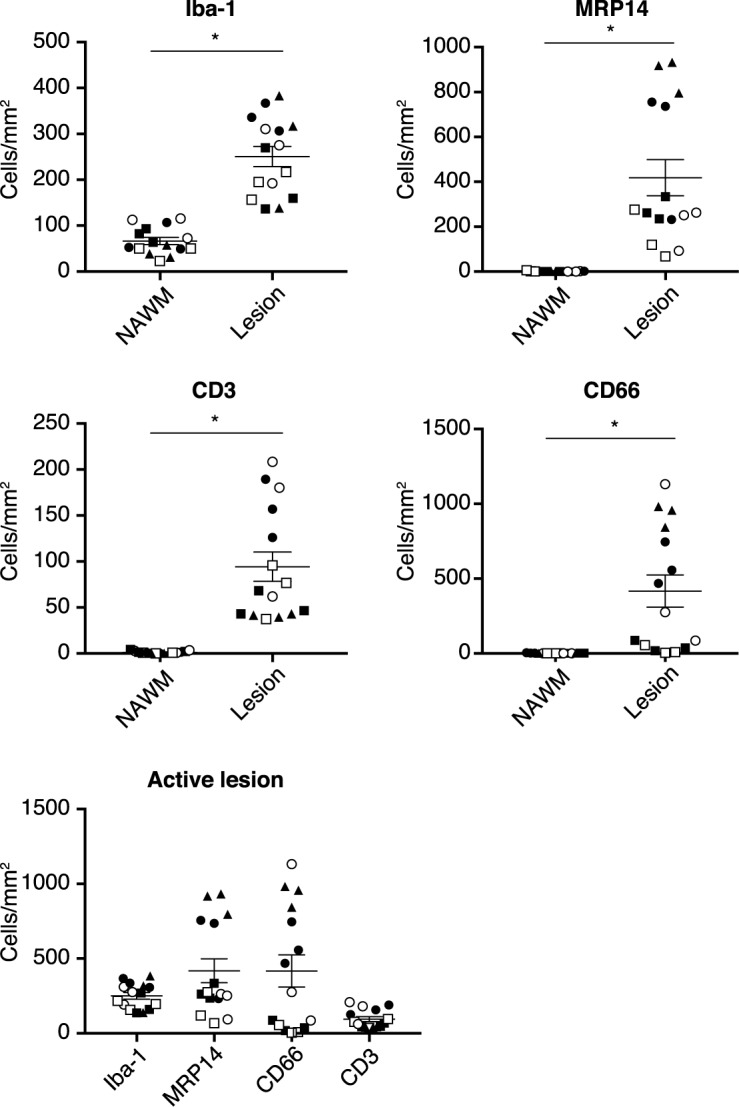
Quantification of lesion cellularity. From 5 monkeys with clinically evident EAE, cell infiltrates in 15 NAWM and 15 active demyelinating areas were quantified with ImageJ and expressed as cells/mm^2^. Shown is the quantification of microglia/macrophage (Iba-1), macrophage and neutrophils (MRP14), neutrophils (CD66) and T cells (CD3) as NAWM versus active lesion (A-D). Lesions of each individual animal are shown by similar symbols: closed circle, R06030; closed square, R06052; open circle, R06088; open square, R07035; closed triangle, R08043. Statistics was calculated with the mean per animal. Results were also combined and reported as cells/mm^2^(F). Statistical significance is indicated as: * when p<0.05.

In the NAWM of the rhesus monkey EAE brain, small clusters composed of neutrophils (CD66), macrophages (myeloid-related protein 14 (MRP14)) and T cells (CD3) were observed (Fig 1). Inflammation and demyelination were found to be widespread and lesion borders of active demyelinating lesions (Fig 1) were not as clearly demarcated as typically observed in the marmoset EAE model. The cellular infiltrate of active lesions is predominantly composed of neutrophils, macrophages and microglia (Fig 1D, 1F and 1H). T cells were substantially detected in the active lesion, albeit less frequent in numbers compared to macrophages and neutrophils (Fig 1J). Quantification of Iba-1, MRP14, CD66, and CD3 was performed in 15 active lesions and 15 areas of NAWM (Fig 2). The number of cells expressing Iba-1, MRP14, CD66, and CD3 were significantly increased in active lesions compared to NAWM, with the highest numbers for macrophages and neutrophils (Fig 2).

### Marked upregulation of oxidative stress markers

**Fig 3:**
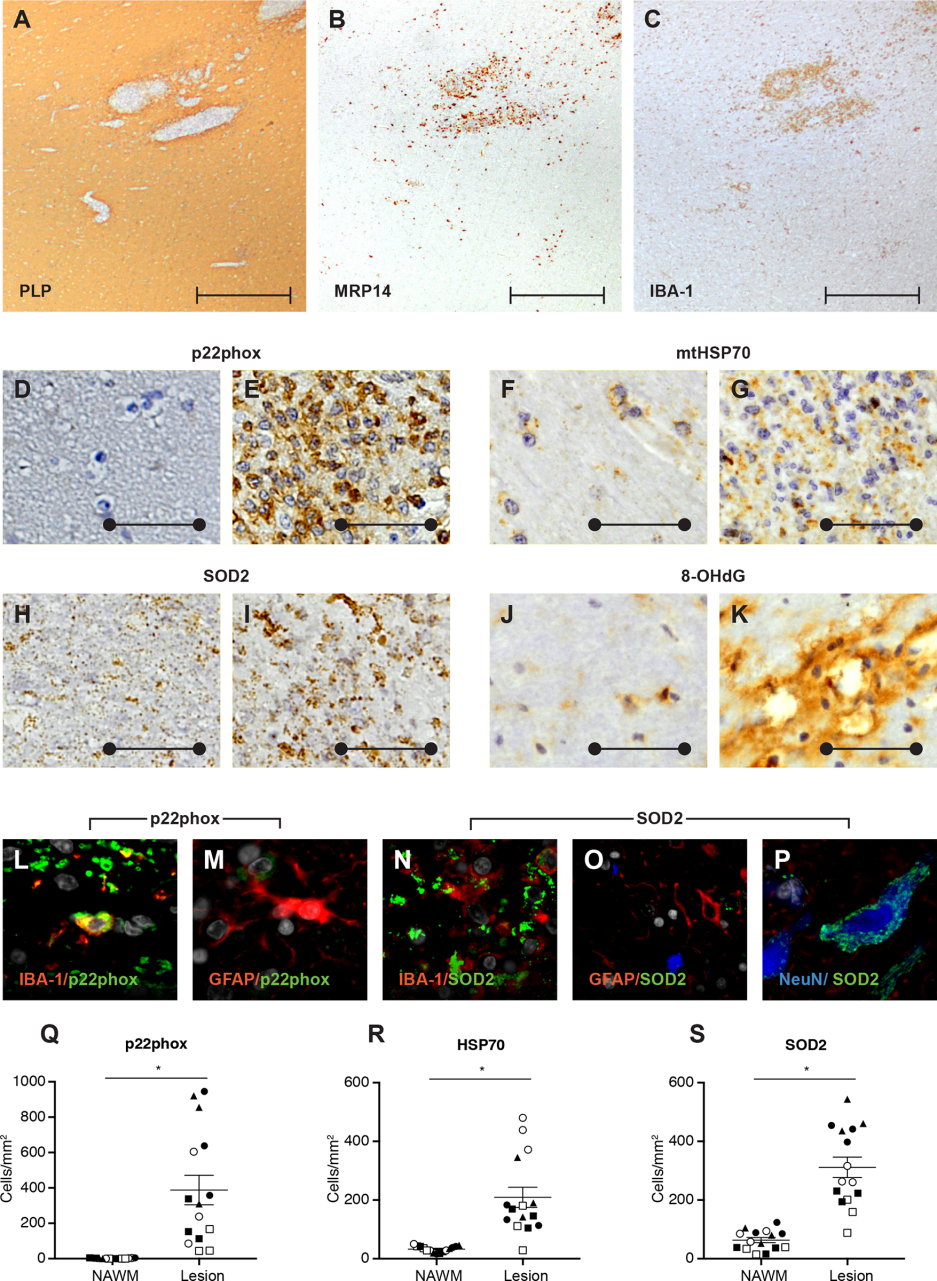
Expression of oxidative stress pathway markers. Expression of key markers of the oxidative stress pathway was analyzed by immunohistochemistry. Shown is a representative brain area with EAE lesions stained for PLP (A), MRP14 (B) and Iba-1 (C). In the NAWM (D), p22phox (D-E, Q) expression ranged from absent to sparse, which contrasted with the marked detection observed in an active lesion (E). Both mtHSP70 (F-G,R) and SOD2 (H-I,S) exhibited basal expression in the NAWM (F, H), while both markers were clearly up-regulated in the active lesion (G,I). Immunoreactivity to 8-OHdG (J-K), a marker of DNA peroxidation, was detected in NAWM as small patches (J), yet markedly upregulated in the active lesion (K). Double labeling of selected oxidative stress markers was performed depending upon host species of primary antibodies. Expression of p22phox (green) was detected in Iba-1+ (red) macrophages and microglia, yet not in GFAP+ astrocytes or other CNS cells (L,M). Expression of SOD2 (green, N-P) was observed in NeuN+ neurons (blue, P) and Iba-1+ (red, N) microglia/macrophages, but fairly absent in GFAP+ astrocytes (red, O). (Q-S) Lesions of each individual animal are shown by similar symbols: closed circle, R06030; closed square, R06052; open circle, R06088; open square, R07035; closed triangle, R08043. Statistics was calculated with the mean per animal. Statistical significance is indicated by * for p< 0.05. Image scale bars are 500 μm (perpendicular line), 50 μm (closed circle).

We examined the presence of oxidative stress mechanisms in the rhesus EAE brain. In NAWM, p22phox, a membrane subunit of NADPH oxidase complex, was sparsely detected, but the expression was strongly enhanced in active lesions (Fig 3A–3E and 3Q). As expected, expression of p22phox was observed in neutrophils as identified by their classic multi-lobe nucleus appearance, and in mononuclear phagocytes, such as Iba-1+ macrophages and microglia, yet not in astrocytes or other CNS glia cells (Fig 3L and 3M).

**Fig 4:**
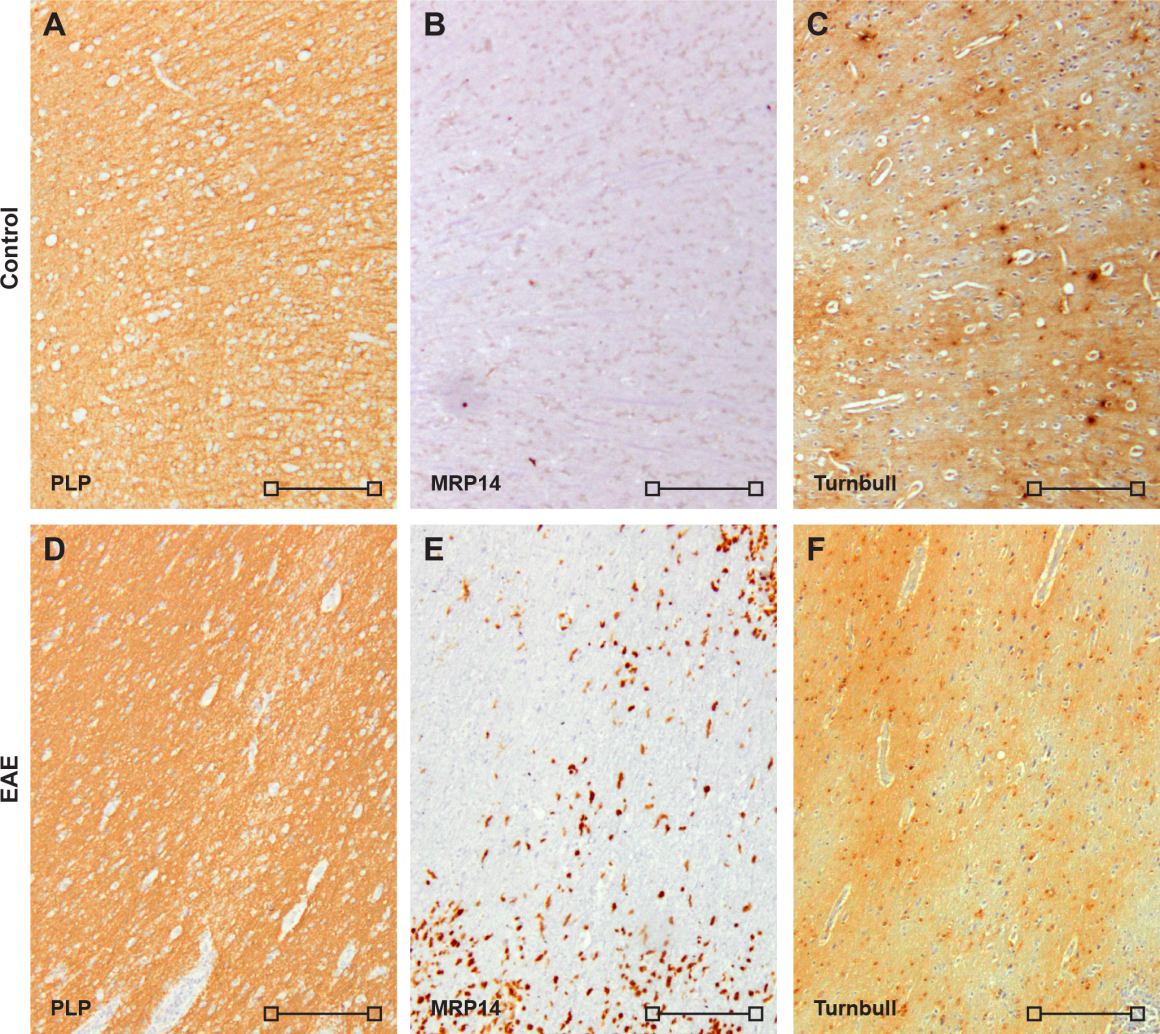
Iron accumulates in rhesus brain. To determine tissue specific iron, Turnbull staining was performed on healthy control and EAE brain tissue from rhesus monkeys. Shown are overview images of PLP (A,D), MRP14 (B,E), and Turnbull (C,F) of a control brain (A-C) and an EAE brain (D-F). In the control brain, iron accumulation was strongest in the GM (A,C). In the EAE brain no distinct pattern of iron staining emerged. Image scale bars are 200 μm (open square).

Basal expression of mtHSP70, a marker of oxidatively stressed mitochondria, was observed in the NAWM of the rhesus monkey EAE brain (Fig 4F and 4R); the expression was markedly up-regulated in the active lesion (Fig 3G and 3R). For a second mitochondrial marker, SOD2, low level expression was observed in NAWM (Fig 3H and 3S) of the rhesus monkey EAE brain and this was significantly upregulated in the active lesion (Fig 3I and 3S). Expression was observed in neurons and Iba-1+ macrophages and microglia, but absent in astrocytes (Fig 3N–3P).

Finally, DNA oxidation, a marker for oxidative injury, was assessed by the presence of immunoreactive 8-OHdG. Small focal patches of immunoreactive 8-OHdG were frequently detected in NAWM, while much larger staining areas were found in the active lesion (Fig 3J and 3K). Collectively this data demonstrates that the oxidative stress pathway is strongly activated in the rhesus monkey EAE brain.

### Accumulation of iron in the rhesus monkey brain

The accumulation of iron in aging human and marmoset brain may contribute to the higher level of oxidative stress in MS and marmoset EAE compared to rodent EAE in which no iron accumulation can be observed. This observation prompted us to stain rhesus control and EAE tissue for total non-heme iron with the Turnbull staining. In the control brain, accumulation of iron could be observed as dark areas in oligodendrocytes and myelin (Fig 4A–4C). Since perivascular lesions varied in demyelination and were not clearly demarcated, distinct iron loss patterns were not clearly observed (Fig 4D–4F), contrasting with observations in MS [29] and marmoset EAE brain [26]. In rhesus EAE brain areas showing inflammation (MRP14) and markers of oxidative stress (p22phox and SOD2), strong perivascular iron staining could be observed, suggesting accumulation of iron in adjacent tissue (S1 Fig).

## Discussion

Induction of the oxidative stress pathway is a key pathological factor in MS [22, 31]. Whereas representation of this pathway in rodent EAE models has shown to be limited, recent data indicate that this pathway is well represented in the marmoset EAE model [26]. Here, we extended these findings to another NHP EAE model, namely in rhesus monkeys. The two models seem to cover the wide spectrum of human autoimmune demyelinating brain diseases, where the chronic EAE model in marmosets resembles MS while the acute EAE model in rhesus monkeys may more closely resemble ADEM [8].

The current histopathological analysis of markers associated with the oxidative stress pathway show that mechanisms associated with the initiation and amplification of oxidative injury are well represented in the acute demyelinating brain lesions in the rhesus monkey EAE model. For both macrophages and neutrophils, the expression of NADPH oxidase and generation of superoxide anions in phagolysosomes play a vital role in pathogen clearance [32, 33]. On the other hand, oxidant production by the NADPH oxidase complex expressed in the cell membrane can be harmful to tissue and result in irreversible oxidative injury. Therefore, healthy tissues are equipped with defense mechanisms against oxidative stress, such as ROS scavengers and glutathione redox cycling. Suppression of this oxidative cytotoxicity without affecting the ROS-dependent intracellular killing of microorganisms is therapeutically attractive for MS [22]. Recent clinical successes of dimethyl-fumarate, which activates the nuclear-related factor 2 (Nrf2) anti-oxidant signaling cascade, suggests cessation of oxidative stress to be clinically beneficial [34].

We observed an increase in lesion-associated cell numbers expressing SOD2, but this may partly be explained by the increase in Iba-1+ cells in a lesion as has been shown in MS [35]. However, SOD2 expression is reflective of oxidatively stressed mitochondria and can thus also be expressed in axons and other CNS cells. Expression of SOD2 was observed in healthy brain neurons as well, but visually this is fundamentally much less than what is seen in the EAE situation. Expression of SOD2 in healthy brain neurons can likely be attributed to its role as a mitochondrial anti-oxidant enzyme; serving a purpose of detoxifying radicals generated from the electron transport chain (ETC). The dramatic increase of SOD2 in neurons of the EAE brain can be attributed to *i)* overall increase in energy demands in diseased brain and thus potentially more free radical production from the ETC; *ii)* underlying inflammation of grey matter (including minor levels in NAGM); or *iii)* oxidative stress due to axon degeneration at distal sites. This observation is consistent with previous reports of enhanced NrF2 expression in Purkinje cells in rodent EAE that is not associated with lesion activity [35].

While the rhesus monkey EAE model diverges from both marmoset EAE and human MS with respect to the histopathological profile of CNS infiltrated immune cells, we propose that there is considerable value of this current model in aiding the development of therapeutics directed towards oxidative injury. Despite the acute nature of the model regarding disease progression, the recruitment of immune cells into the CNS elicited strong representation of the oxidative stress pathway. As rhesus monkey EAE would represent the extreme end of acute oxidative stress in relation to CNS demyelination, therapies showing efficacy via anti-oxidant stimulation in this model, would have a good chance at working in MS.

Primate models of autoimmune demyelination demonstrate tremendous heterogeneity with respect to clinical and neuropathological presentation and thus represent a wide spectrum of inflammatory associated demyelination [8, 36]. The observation that entirely different clinical and pathological processes occur in different primate species indicates an important role of innate factors in the clinical and pathological response to immunization [8, 9]. It is tempting to speculate that the heterogeneous disease course in human demyelinating disorders and in primates is a function of differential activation of innate immune factors, neutrophils in particular, and the role that oxidative stress plays [9]. Moreover, tissue defense mechanisms may differ between species, as shown for the complement regulatory factor CD55 [37]. Although the T and B cells remain the center of attention for immune modulatory therapies, selective targeting of the innate immunity may prove to be successful as suggested by others [38].

The accumulation of iron in the rhesus monkey brain is in line with earlier observations that iron accumulates in myelin and oligodendrocytes of the marmoset brain [26]. The results reported here are also consistent with reports on presence of iron in the human brain, suggesting that CNS accumulation of iron is a general neurophysiological feature of humans and NHP and represents a critical divergent from rodent species [27]. Age-associated accumulation of iron in the human brain is thought to play a critical role in the amplification of oxidative damage in neurodegenerative processes via the Haber-Weiss reaction [39]. Pathology-associated liberation of iron could enhance the oxidative stress elicited by the respiratory bursts of neutrophils and macrophages [39]. Indeed, there is mounting correlative evidence that links iron and oxidative stress in the MS brain [40]. Thus, pathology specific iron liberation, hypothetically, could enhance oxidative stress observed in the rhesus EAE brain; however, the lack of clearly demarcated demyelination in this model make correlating iron staining patterns to oxidative stress patterns difficult. As iron is vital for the re-myelination process, yet may also contribute to oxidative injury, future investigations using NHP EAE models can provide clarity on the role of iron in demyelinating pathologies [19].

In conclusion, we demonstrate that the rhesus monkey EAE brain, like the marmoset EAE brain, replicates many key features of the oxidative stress pathway observed in human CNS demyelinating disorders. Both NHP EAE models can therefore be beneficial in validating therapy targets for pathogenic mechanisms associated with oxidative injury.

## Supporting information

S1 Fig Iron and oxidative stress. Shown are adjacent stains of PLP (A), MRP14 (B), p22phox (C), SOD2 (D) and Iron (E) of an EAE lesion. The image scale bar is 100 μm.
(TIF)

## Abbreviations

ADEM: acute disseminated encephalomyelitis
CNS: central nervous system
DAB: diaminobenzidine tetrachloride
EAE: experimental autoimmune encephalomyelitis
(rh)MOG: (recombinant human) myelin oligodendrocyte glycoprotein
MRP14: myeloid-related protein 14
MS: multiple sclerosis
mtHSP70: mitochondrial heat shock protein 70
NAWM: normal appearing white matter
NHP: non-human primate
PLP: proteolipid protein
ROS: reactive oxygen species
SOD: superoxide dismutase
