# Supplementary figures and images for: Severe oxidative stress in an acute inflammatory demyelinating model in the rhesus monkey
Source: PLoS One. 2017 Nov 14;12(11):e0188013. doi: 10.1371/journal.pone.0188013 (PMC5685592; doi:10.1371/journal.pone.0188013)

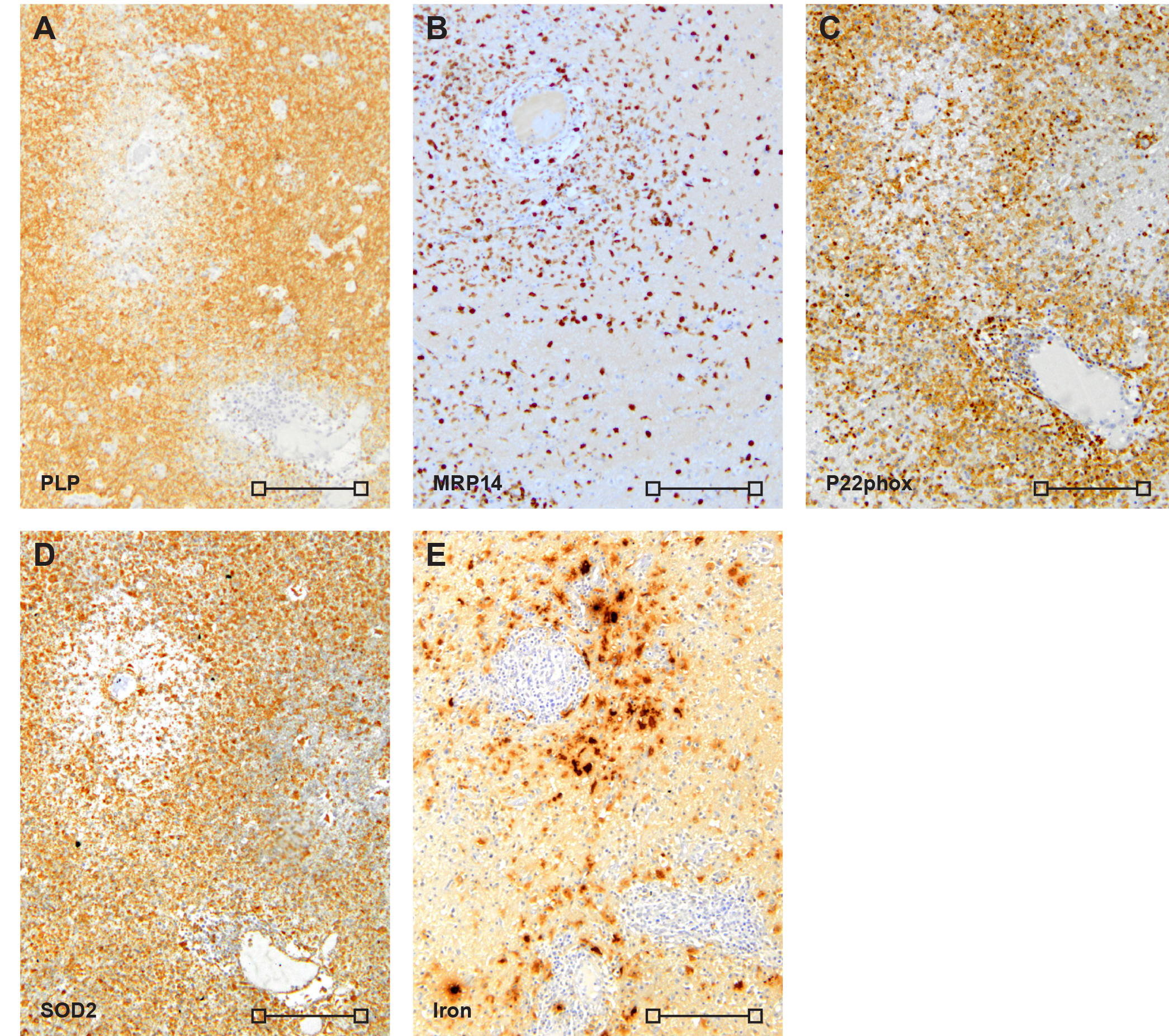

Supplement: S1 Fig — Shown are adjacent stains of PLP (A), MRP14 (B), p22phox (C), SOD2 (D) and Iron (E) of an EAE lesion. The image scale bar is 100 μm. (TIF) [file pone.0188013.s001.tif]
